# Oxidative Crosslinking of Peptides and Proteins: Mechanisms of Formation, Detection, Characterization and Quantification

**DOI:** 10.3390/molecules27010015

**Published:** 2021-12-21

**Authors:** Eduardo Fuentes-Lemus, Per Hägglund, Camilo López-Alarcón, Michael J. Davies

**Affiliations:** 1 Department of Biomedical Sciences, Panum Institute, University of Copenhagen, 2200 Copenhagen, Denmark; eduardo.lemus@sund.ku.dk (E.F.-L.); pmh@sund.ku.dk (P.H.); 2 Departamento de Química Física, Facultad de Química y de Farmacia, Pontificia Universidad Catolica de Chile, Santiago 7820436, Chile; clopezr@uc.cl

**Keywords:** crosslink, dimerization, protein oxidation, radicals, di-tyrosine, di-tryptophan, disulfides, thiols, aggregation, proteomics, mass spectrometry

## Abstract

Covalent crosslinks within or between proteins play a key role in determining the structure and function of proteins. Some of these are formed intentionally by either enzymatic or molecular reactions and are critical to normal physiological function. Others are generated as a consequence of exposure to oxidants (radicals, excited states or two-electron species) and other endogenous or external stimuli, or as a result of the actions of a number of enzymes (e.g., oxidases and peroxidases). Increasing evidence indicates that the accumulation of unwanted crosslinks, as is seen in ageing and multiple pathologies, has adverse effects on biological function. In this article, we review the spectrum of crosslinks, both reducible and non-reducible, currently known to be formed on proteins; the mechanisms of their formation; and experimental approaches to the detection, identification and characterization of these species.

## 1. Introduction

The formation of covalently linked peptides and proteins plays a key role in many biological processes, both physiologically and pathologically. These can be formed intentionally, such as in the oxidative folding of nascent proteins within mammalian cells in the endoplasmic reticulum or Golgi involving the generation of disulfide bonds from two cysteine (Cys) residues and in the assembly of insect exoskeletons via the crosslinking of two tyrosine (Tyr) residues, or as a result of accidental exposure to oxidizing species (low-molecular mass or enzymes) that chemically link two protein sites. These crosslinks can be formed between different sites within the same molecule (intramolecular or intrachain crosslinks), between two different chains in a single molecule (e.g., the interchain crosslinks in mammalian insulins), or between two separate species (intermolecular crosslinks). Some of these crosslinks play a key role in stabilizing or maintaining proteins structures and can be essential to functional activity [1], whereas others have negative effects of biological function (e.g., altered turnover, lifetime or activity) [2]. Whilst some crosslinks appear to be benign and devoid of adverse effects and end up as targets of catabolic processes (e.g., degradation by proteasomes, lysosomes, other proteases), others are strongly associated with adverse effects and are implicated (in some cases, causally) in the development of pathologies (e.g., [3,4]).

Whilst it is well established that crosslink formation can have major effects on biological systems—either positively or negatively—our knowledge of the full complement of crosslinks formed in biological systems (the ‘crosslink-ome’, ‘X-link-ome’) is far from complete [2]. The number of disulfide-containing proteins is large and relatively well defined, with these being particularly abundant in proteins found in biological fluids (e.g., human serum albumin in plasma contains 17 disulfides), in structural proteins such as receptors (e.g., the low-density lipoprotein receptor, LDLR, contains 30 unique intradomain disulfides [5]) and extracellular matrix proteins (e.g., laminin contains ~200 disulfides). Disulfides are also present at more modest levels in intracellular proteins, where they can function as structural elements, allosteric effectors, or be involved in catalytic cycles [1,6]. For proteins containing many disulfides, ensuring correct pairing of Cys residues into disulfides during synthesis and assembly is a complex problem [7].

Unlike the relatively well-characterized complement of disulfide-containing proteins, the number, occurrence and sites of other potential crosslinks is poorly understood, but the subject of active research. Recent developments in our understanding of the chemistry of crosslink formation, and the development of new and more sensitive methods for their detection is driving advances in this field. This article summarizes recent developments, with a focus on crosslinks formed by oxidation (either enzyme-mediated or via the reactions of low-molecular-mass oxidants). Crosslinks formed by deliberately added reagents (e.g., dicarbonyl and related crosslinking agents, such as glutaraldehyde) or those arising from glycation/glycoxidation reactions of sugars are not discussed for reasons of space. These have been discussed elsewhere [8,9,10].

## 2. Enzymatic Protein Crosslinking

Multiple enzymes can mediate the crosslinking of proteins, with a few key examples briefly summarized below. Enzyme-generated crosslinks are critical to the formation of many three-dimensional structures as these provide strength and rigidity, if biologically required. Examples include crosslinks formed within the extracellular matrix (ECM) of most, if not all, tissues, such as those formed between matrix proteins, and particularly collagens by the copper-containing lysyl oxidase (LOX) and LOX-like (LOXL) enzymes (reviewed in [11]). LOX oxidizes specific lysine (Lys) and hydroxylysine residues to carbonyls that undergo subsequent reactions to crosslink collagens (e.g., types I and III) and elastin [11,12,13,14]. In contrast, the LOXL family of enzymes acts on collagen type IV and drives the assembly of basement membranes [11,15]. Other enzymes also contribute to collagen crosslinking in the ECM with peroxidasin, a member of the heme peroxidase superfamily, mediating the formation of highly specific methionine (Met) to Lys crosslinks within the NC1 domains on collagen via generation of the oxidant hypobromous acid (HOBr). This species reacts rapidly with the Met residue to form an intermediate that then reacts with a suitably positioned Lys residue [16,17] (see also below). This type of crosslinking has been reported across many species [18]. Other members of the peroxidase superfamilies (e.g., horseradish peroxidase, myeloperoxidase, laccase) can also generate crosslinks via enzyme-mediated oxidation of substrates to radicals which then undergo radical–radical coupling. A classic example is oxidative coupling of Tyr and a wide range of other phenols via phenoxyl radical generation [19,20,21].

Isopeptide crosslinks involving reactions of the Lys side-chain amine with the carboxylic acid of aspartic acid (Asp), or the amides of asparagine (Asn) and glutamine (Gln) residues can be formed spontaneously or enzymatically [22,23,24]. These can be formed intracellularly, or within the ECM, with their function being associated in the latter case not only with enhanced ECM rigidity, but also in the attachment of bacterial pathogens to host tissue collagens and fibrinogens [25]. Enzymatic isopeptide bonds, formed by factor XIIIa (FXIIIa) between Lys and Gln residues, are critical to the formation of fibrin assemblies in blood clotting. Related crosslinks are generated by tissue transglutaminase enzymes [26]. These crosslinks are used to attach targeting moieties to specific proteins, including ubiquitin to proteins destined for proteosomal degradation [27], and small-ubiquitin-like modifier proteins (SUMOs, via the C-terminal glycine, Gly, residue) in the case of protein transport, targeting and regulation. These crosslinks involve the initial activation of the ubiquitin/SUMO, and subsequent transfer to the target, involving multiple activating (e.g., E1), conjugating (E2) and ligation (E3) enzymes [28]. Multiple glutamic, Glu and Gly residues have also been detected attached to Glu residues in the C-terminal regions of microtubulin, with this reported to be involved in microtubule function [29].

Covalent bonds are also widely and deliberately generated between proteins and co-factors to produce functional enzymes, with examples including the crosslinking of heme, flavin, pyridoxal, biotin, thiamine, molybdopterin and lipoic acid to proteins (see, for example, [30]). Whilst of considerable interest and importance, further discussion of these reactions lies outside the scope of this review, and subsequent sections are focused on oxidant-mediated reactions, though some of these enzyme-mediated crosslinks involve the formation and reactions of oxidants (e.g., by peroxidases and peroxidasin).

**Figure 1:**
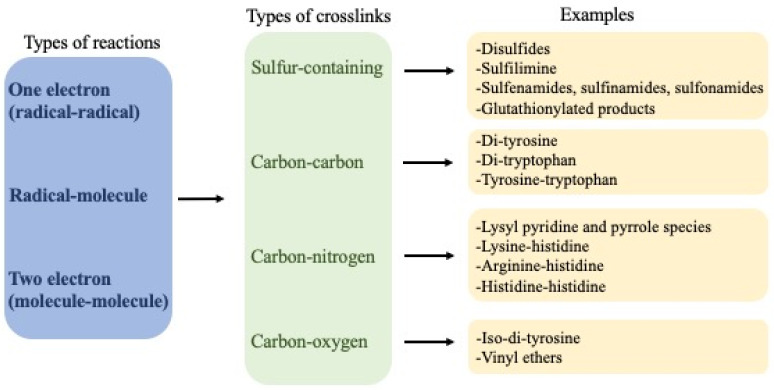
Overview of crosslinks formed on proteins, their nature and mechanisms of formation.

An overview of the crosslinks that are discussed further in this review is presented in Figure 1.

## 3. One-Electron (Radical–Radical) Reactions

Dimerization of two radicals to form a new covalent bond is typically a very fast process due to the low energy barriers for such reactions. Therefore, they are a major source of crosslinks in peptides and proteins when the radical flux is high and there are limited competing reactions. Most carbon-centered protein radicals (P^•^) formed from aliphatic side-chains by hydrogen–atom abstraction reactions react rapidly with O_2_ at diffusion-controlled rates (k ~ 10^9^ M^−1^ s^−1^) to give peptide or protein peroxyl radicals (P-OO^•^) [31]. The rapidity of these reactions limits direct reactions of two P^•^**,** except in circumstances where the O_2_ concentration is low. This is of biological relevance, as hypoxia is a common phenomenon, with endogenous levels of O_2_ being typically in the range 3–70 μM [32]. However, lower concentrations are present in situations where demand is great (e.g., high metabolic rates) or perfusion is poor (e.g., in the core of many solid tumors), thereby limiting P-OO^•^ formation and allowing (P-P) dimer formation [33]. For the limited number of P^•^, where reaction with O_2_ is slow or modest, as is the case for Cys-derived thiyl radicals (RS^•^, *k* < 10^7^ M^−1^ s^−1^ [34]), tryptophan (Trp) indolyl radicals (Trp^•^, *k* < 4 × 10^6^ M^−1^ s^−1^ [35,36]) and Tyr phenoxyl radicals (Tyr^•^, *k* < 10^3^ M^−1^ s^−1^ [37]), formation of disulfides (cystine) from two RS^•^, di-tyrosine from two Tyr^•^, di-tryptophan from two Trp^•^, and crossed dimers between these (e.g., Tyr–Trp) can be generated. The structure and mechanisms of the formation of these species are discussed below.

Light, particularly of wavelengths >~280 nm, which are not absorbed by the ozone layer, can penetrate significantly into biological structures and be absorbed either directly by protein residues, particularly Trp, Tyr and cystine [38], or by other species with high extinction coefficients in the long wavelength UV or visible regions. Energy absorption by non-protein species can give rise to indirect protein oxidation via the formation of excited states (e.g., singlet oxygen, ^1^O_2_ and reactive triplets) and/or radicals [38]. Direct UV absorption by proteins can form RS^•^ from homolysis of the –S–S– bond of cystine (with C-S cleavage being an alternative pathway), and Tyr and Trp radicals by photo-ionization of these side-chains. These species can then give rise to crosslinks.

## 4. Radical–Molecule Reactions

Radical–molecule reactions appear to be a limited pathway for the formation of protein crosslinks, due to the absence of double bonds to which radicals might add in proteins, and limited stability of adducts to aromatic rings. Notable exceptions are the rare amino acids dehydroalanine (DHA; 2-aminoacrylic acid) and dehydroaminobutyric acid (DHB; 2-aminocrotonic acid). These contain a double bond between the α- and β-carbons of the side-chain and are non-proteinogenic species [39], with these being generated via elimination reactions of serine residues (Ser), phospho-Ser and selenocysteine (Sec) residues (in the case of DHA) [39], and from threonine (Thr) and phospho-Thr (in the case of DHB) [40]. DHA can also be formed via cleavage of the carbon–sulfur bonds of the disulfide cystine, via mechanisms involving RS^•^ or nucleophilic elimination reactions [39].

Although radical addition to double bonds is typically rapid and energetically favorable due to low energy barriers, these reactions are rare as the concentrations of both DHA and DHB (with the former more abundant) and the radicals that might undergo addition with them are very low. Nevertheless, some examples are known for radicals that have relatively long lifetimes and modest rates of reaction with O_2_ (i.e., Cys thiyl, Tyr phenoxyl, Trp indolyl) [41].

## 5. Two-Electron (Molecule–Molecule) Reactions

Reactions between two molecules are typically much slower than between two radicals or radical–molecule reactions. However, the concentration of the reactants is often much higher than for reactive intermediates, and consequently, the overall rates of these reactions may be significant—and the yield of products greater—than for the processes outlined above. These reactions are therefore major sources of protein crosslinks. The rate constants for these reactions would be expected to vary enormously—though quantitative data is lacking for most systems—with some reactions involving unstable species (e.g., sulfenic acids (RSOH), S-nitrosothiols (RSNO), unsaturated aldehydes/ketones, quinones) being relatively rapid (i.e., occurring over seconds/minutes).

## 6. Types of Crosslinks Detected within and between Proteins and Peptides

The following sections and Table 1 summarize various types of crosslinks that have been detected within and between peptides and proteins, the nature of these species, their reversibility, mechanisms of formation and, subsequently, methods available to detect, identify, characterize and quantify these species.

### 6.1. Sulfur-Containing Crosslinks

#### 6.1.1. Sulfur–Sulfur Crosslinks (Disulfides)

Sulfur–sulfur crosslinks are the most common form of crosslinks present in biological systems, generated by multiple enzymatic and non-enzymatic pathways. Many endogenous (native) disulfides are formed during or shortly after protein synthesis in the endoplasmic reticulum or Golgi, via enzyme-guided reactions. During this process, erroneous (incorrect) disulfides appear to be formed to a significant extent, as judged by the presence of multiple enzymes/repair systems that allow reshuffling of incorrect linkages (e.g., [7,88]). These processes have been widely researched and are not discussed further here.

**Figure 2:**
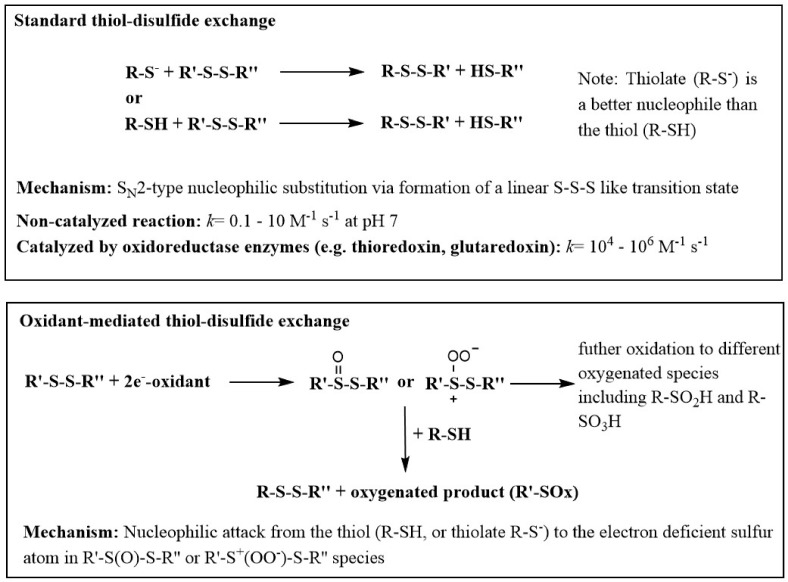
Mechanisms of traditional ‘thiol–disulfide exchange’ (top) and ‘oxidant-mediated thiol–disulfide exchange’ (bottom) reactions.

Multiple other pathways can also generate non-intended disulfides, including thiol–disulfide exchange reactions in which a thiolate anion (RS^−^, the ionized and more reactive form of a thiol) reacts with a disulfide, with the formation of a new disulfide and release of a thiol [89] (Figure 2, top section). These reactions are typically slow, as there is little thermodynamic driving force for the reaction, but they can occur at significant rates if the original disulfide is subject to significant strain that is released on reaction with the incoming RS^−^ [89,90]. The rate of these reactions can also be enhanced, to a very significant extent, within the active sites of enzymes, with di-thiol/disulfide cycles being a common feature of a number of important proteins (e.g., thioredoxins [91]).

Rapid formation of new disulfide bonds has also been reported in so-called ‘oxidant-mediated thiol–disulfide exchange reactions’, where the original disulfide is initially oxidized, by a range of different species, to a more reactive intermediate (e.g., a thiosulfinate or peroxidic intermediate) that subsequently undergoes rapid reaction with another RS^−^ [92,93,94] (Figure 2, lower section). This type of process gives rise to glutathione (and other thiol) adducts to disulfide-containing peptides and proteins (i.e., glutathionylated species [92]), and also new (intermolecular) protein–protein crosslinks [93,94]. In some cases, the residues involved and the exact sites of the new disulfides have been determined by LC-MS peptide mass mapping [93,94] (see also below). The more rapid rate of these reactions relative to ‘native’ thiol–disulfide exchange arises from the activation of the disulfide via the initial oxidation, which typically involves the formation of an electron-deficient center.

**Figure 3:**
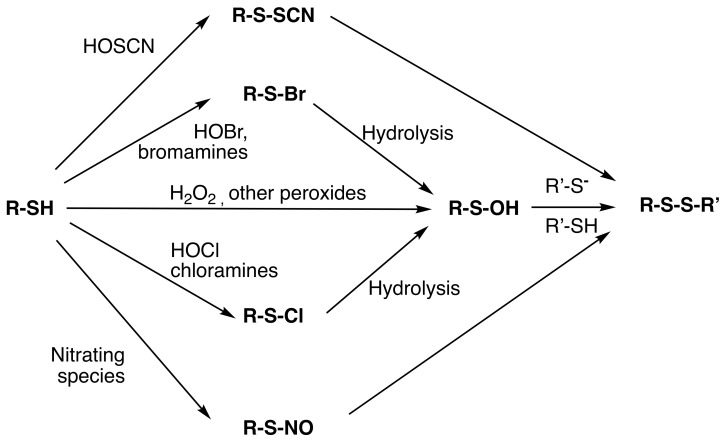
Generation of crosslinks via oxidized thiol residues. Similar reactions of the ‘activated’ thiols (RS–OH, RS–Cl, RS–Br, RS–SCN, RS–NO) can occur with nitrogen nucleophiles (e.g., RNH_2_) to give new S–N bonded species (see text for further details).

New disulfides can also be formed from thiols via a similar activation (oxidation) process. Thus, reaction of thiols with H_2_O_2_, HOCl, HOBr, HOSCN, HNO and ONOOH (amongst others) yields short-lived intermediates that undergo subsequent rapid reaction with another RS^−^/RSH to give a disulfide (Figure 3). These reactions are facile and rapid in most cases as the displaced group is a stable anion (e.g., HO^−^, Cl^−^, Br^−^, etc.). These are very well established with RS-OH (sulfenic acids) and RS-NO [95], but these are some of the least reactive members of this family, as Cl^−^ and Br^−^ are, for example, much better leaving groups than HO^−^. Indeed, isolation of RS-Cl, RS-Br and RS-SCN species is difficult due to their high reactivity with nucleophiles, including water. In the latter case, hydrolysis gives RS-OH, which undergoes similar reactions. Some RS-OH and RS-NO species are sufficiently long-lived to be detectable on proteins, though their lifetimes are structure- and environment-dependent [96].

Disulfides can also be formed from rapid termination reactions of two RS^•^. However, reaction of RS^•^ with another R’S^−^ to give the corresponding disulfide radical anion (RSS’R^•−^) is also very rapid, and as the concentration of R’S^−^ (either on a protein, or a low molecular mass species such as glutathione) is typically much higher than that of RS^•^, the radical anion pathway usually predominates. The resulting RSS’R^•−^ undergoes rapid electron transfer with electron acceptors, including O_2_, resulting in the formation of a new disulfide (which may be either a protein–protein species, or a glutathionylated protein) and the superoxide radical anion, O_2_^•−^.

#### 6.1.2. Sulfur–Nitrogen (S–N) Crosslinks (Sulfimines, Sulfenamides, Sulfinamides and Sulfonamides)

A wide range of S–N crosslinks are known, with these arising predominantly from molecule reactions of activated sulfur centers with nitrogen nucleophiles, and particularly the side-chain amine of Lys (or hydroxy-Lys) residues (see also Figure 3). Reactions with other nitrogen nucleophiles are known, including the more reactive (when compared to the Lys side-chain) N-terminal amine of peptides or proteins. Reactions at (backbone) amide and guanidine (Arg side-chain) functions have also been reported (see below), but such examples are limited, as these are weaker nucleophiles.

Crosslinking can occur via the thioether group of methionine in the enzymatic (peroxidasin)-induced crosslinking of the NC1 domains of collagens. Reaction of a specific Met residue (Met^93^) with HOBr formed by peroxidasin generates a transient bromosulfonium ion [–S(Br)^+^–] that reacts with the amine group of a suitably positioned hydroxy-Lys residue (Hyl^211^) to give an interchain sulfilimine (–S=N–) crosslink. These crosslinks are critical for the generation of functional extracellular matrices in many organisms, with an absence of this enzymatic activity causing tissue dysfunction that is embryonically lethal in some species [16,17]. Reaction with the amine function occurs in competition with the reaction with water (acting as a nucleophile), with consequent methionine sulfoxide formation. This type of crosslink is not unique to peroxidasin-generated HOBr, with other Met-containing peptides undergoing similar sulfilimine crosslink formation with Lys residues, either intra- or intermolecularly [97]. These reactions are also not limited to HOBr, with HOCl, bromamines and chloramines (RNHBr and RNHCl species, respectively) also generating these species in competition with the sulfoxide [97]. In the case of HOCl, these species are usually formed in low yield. For the chloramines, initial oxidation of the sulfur center of the Met to give the chlorosulfonium ion [–S(Cl)^+^–] appears to be followed by rapid reaction with the nitrogen atom of the original chloramine [97], indicating that these reactions can be highly specific, and that the [–S(Cl)^+^–] species is short lived.

Related reactions of sulfenyl halides (RS–X) and related species formed from reaction of the –SH group of Cys or GSH with an oxidant (e.g., HOCl, HOBr, chloramines, bromamines, ONOOH) with amine nucleophiles have been reported to give rise to a large family of sulfenamides (RS–NHR’), sulfinamides [RS(O)–NHR’] and sulfonamides [RS(O)_2_–NHR’]. These have been detected as both intra- and intermolecular crosslinks in both peptides and proteins [98,99,100]. A well-established example is glutathione sulfonamide (GSA) formation, which has been used as a biomarker of oxidative damage in both cells (e.g., [101,102]) and human tissues and fluids [103,104]. In this case, the linkage is formed intramolecularly with the amide nitrogen of the terminal Gly residue, resulting in an eight-membered ring species [105]. The formation of GSA occurs in competition with reaction of the RS-X with another RSH to give the disulfide (i.e., GSSG in the case of oxidation of GSH) with the ratio of GSA:GSSG dependent on the oxidant [98]. The disulfide is usually the major product, but the GSA yield can be high in some cases [98,105], including in some cells [101,102,106]. The formation of GSA requires three equivalents of oxidant, and the exact sequence of oxidation events is unclear (i.e., whether oxygenation at the sulfur occurs prior to, or after the formation of the S–N bond) [105]. The detection of sulfenamides and sulfinamides in other systems (e.g., S100A8 proteins [99] and peptides [100,107]) suggests that S–N bond formation may be the initial event, with oxidation at the sulfur occurring on reaction with the second and third oxidizing equivalents. These species are poorly reducible when compared to disulfides [99], and only ~50% of the GSH oxidized by HOCl is recovered (presumably from GSSG) on reduction [108]. Evidence has also been reported for model peptides for the involvement of the guanidine group of Arg residues in the formation of S–N crosslinks [100].

There is considerable evidence of the formation of an intramolecular sulfenamide (sulfenyl amide, S–N species) in the enzyme protein tyrosine phosphatase 1B (PTP1B), with the linkage formed between the sulfenic acid (RS–OH) form of the catalytic Cys residue, and the adjacent main chain amide of a Ser residue [46]. This modification appears to protect the Cys residue from irreversible oxidation to sulfinic (RSO_2_H) or sulfonic (RSO_3_H) acids and loss of enzyme activity. This species appears to be the stable, inactive ‘resting state’ form of the enzyme, with the sulfenyl amide being readily reversed by cellular thiols such as GSH, with conversion back to its catalytically active Cys form [46].

#### 6.1.3. Sulfur–Carbon (S–C) Crosslinks

Sulfur–carbon crosslinks can be generated via the addition of RS^•^ (radical addition) or RS^−^ (Michael adduction) to the double bonds of DHA and DHB. These are likely to be minor pathways in biological samples due to the low concentrations of DHA/DHB and the occurrence of other alternative reactions of RS^•^ (see above), though these reactions have been detected with some isolated proteins [109] and also in the nucleus of the lens [63].

**Figure 4:**
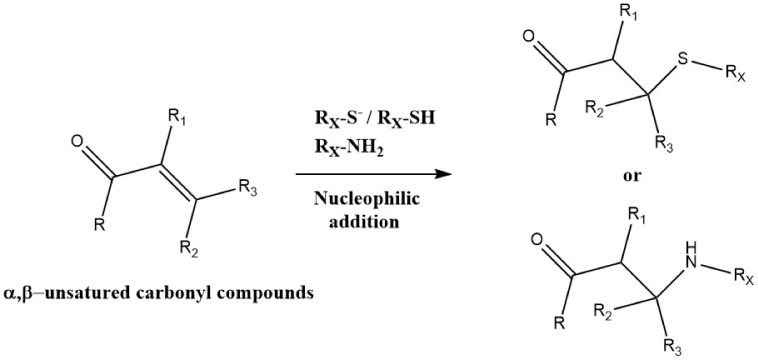
Michael addition reactions of nucleophiles to αβ-unsaturated carbonyl compounds.

Related Michael addition reactions of RS^−^ with quinones, or αβ-unsaturated aldehydes and related species yield S-C bonds (e.g., [110,111,112] (Figure 4). The rate constants for these addition reactions are fast for some quinones (*k* 10^2^ − 10^5^ M^−1^ s^−1^ for 1,4-benzoquinone, with lower values for more sterically hindered species [110]), but slower for αβ-unsaturated aldehydes and related species (*k* < 1 − 7 × 10^2^ M^−1^ s^−1^ [111]). As expected, the rate constants vary significantly with the structure of the Michael acceptor (quinone/aldehyde), and in the majority of cases with the thiol pK_a_ (i.e., higher concentrations of the better nucleophile RS^−^) [110,111]. Some of these reactions are reversible, with another RS^−^ able to displace the added quinone [113]. Whilst these reactions do not directly give protein–protein crosslinks, the added quinone can undergo further reaction with another RS^−^ at the second double bond, resulting in protein–protein linkages via the original quinone. These reactions are responsible for some of the crosslinking detected in aged and cataractous lenses, with the quinone species being derived from Trp metabolites. These Trp-derived species act as photophysical filters that protect against UV in young lenses but become increasingly oxidized and adducted to lens crystallin proteins with increasing age and light exposure [114].

Similar reactions can occur with quinones generated on proteins via oxidation of Tyr and Trp, though the latter are less well characterized. Thus, radical and photo-oxidation reactions can convert Tyr to 3,4-hydroxyphenylalanine (DOPA), with this undergoing rapid subsequent oxidation to the quinone (DOPA quinone). This quinone is then susceptible to attack by RS^−^ on peptides (e.g., GSH) or other proteins, via Michael addition, with this giving glutathionylated proteins or protein–protein crosslinks. These types of reactions have been reported for model peptides and myoglobin subject to oxidative damage [42] and with casein proteins subjected to photo-oxidation (Rossi et al., submitted). Similar reactions can occur within the active site of some enzymes (e.g., copper amine oxidases [115]) where GSH appears to modify the cofactor, which is a redox-active hydroxylated/quinone Tyr or Trp species, possibly via addition [116]. The co-factor in a number of dehydrogenases and oxidase enzymes is cysteine tryptophanylquinone (CTQ), which involves a linkage between Cys residues and an oxygenated Trp [117]—this appears to be formed via initial Trp oxidation in a complex series of enzyme-mediated reactions and subsequent Michael adduction of the Cys to the modified Trp. Related tryptophan tryptophanylquinone cofactors (TTQ), containing Trp-oxygenated Trp crosslinks, are present in selected aliphatic and aromatic amine dehydrogenase enzymes [118].

Evidence has been presented for a radical-mediated crosslink between Tyr^A19^ and Cys^B20^ in photo-oxidized insulin. Irradiation of the protein generates RS^•^ by direct homolysis of one of the three disulfides present in this protein [119]. Subsequent electron/hydrogen transfer from Tyr to one of the RS^•^ would then give the required radical pair for formation of the dimer. Alternatively, photo-induced electron transfer from a photo-excited Tyr to a disulfide could occur, with this yielding Tyr^•^ and a single RS^•^, with the crosslink being formed by subsequent reaction between these two species. A third potential pathway may involve *addition* of RS^•^ formed from Cys^B20^ radical to the intact Tyr^A19^ residue followed by oxidation of the radical adduct [119,120]. The exact structure of the adduct species (i.e., whether this involves an S–O or S–C bond) remains to be resolved. In addition to this Tyr–Cys crosslink, a dithiohemiacetal [–C(SH)–S–C–] crosslink was also detected, involving Cys^A20^ and Cys^B19^, on photolysis of human insulin in the solid state [121]. The proposed mechanism involves formation of two RS^•^ from the disulfide, followed by disproportionation of the two radicals to give a thiol (RSH) and a thioaldehyde (–C=S), and then molecular addition of the thiol to the thioaldehyde. Similar species have been detected with other photo-oxidized peptides/proteins, including mouse and human growth hormones and some monoclonal antibodies (reviewed in [120]). A variant on the above reaction has been detected when Ser residues are located in the vicinity of the thioaldehyde, with the hydroxyl group of the Ser reacting with the thioaldehyde to give a thiohemiacetal [–C(SH)–O–C–] [41]. Subsequent cleavage of the C–S bond under continued irradiation, and loss of the sulfur center via homolytic or heterolytic reactions, results in a vinylether crosslink (–C=C–O–C) [41]. It is likely that similar reactions occur with Thr residues, and analogous reactions have been detected with a Tyr in place of the Ser (reviewed in [120]).

A complex crosslink involving a nitrogen–oxygen–sulfur (N–O–S) linkage has been reported between a Lys and Cys in a transaldolase reductase from *Neisseria gonorrhoeae*, the pathogen that causes gonorrhea [45]. Interestingly, this crosslink regulates enzyme activity via an allosteric switch, suggesting that its formation may be of biological importance. Whilst the structure of the crosslink has been determined in detail (by X-ray crystallography and others), the mechanism of its formation is unresolved. Multiple mechanisms have been proposed, with each involving initial oxidation at the sulfur to give oxygenated products [45].

#### 6.1.4. Sulfur–Selenium Crosslinks

As sulfur and selenium have considerable similarities in their chemistry, there is evidence for intra- and intermolecular selenium–sulfur and selenium–nitrogen species. Examples of both types are found in two of the major families of enzymes that protect cells against oxidative stress: the glutathione peroxidases (GPx’s), which remove H_2_O_2_ (and lipid hydroperoxides in the case of GPx4), and the thioredoxin reductase (TrxR)/thioredoxin (Trx) redox systems, that maintains thiols in their reduced form. In order to rationalize the resistance of GPx enzymes to deselenation (loss of selenium to give DHA), it has been proposed that internal selenenyl amide (Se–N) linkages with neighboring amides involving five- or eight-membered rings [122] are formed during the catalytic cycle [123]. These selenenyl amides subsequently react with GSH to give a selenenyl GSH (i.e., Se–S) species, which is then repaired at the expense of GSH.

Similar Se–S species are generated during the catalytic cycle of the TrxR/Trx redox system. The resting state of TxrR contains an intramolecular Se–S bond between a Cys and Sec residue, which is reduced by NADPH via an FAD cofactor. The resulting selenate anion (RSe^−^) then reacts with the intramolecular disulfide bond of oxidized thioredoxin to give an intermolecular Se–S linkage between the TrxR and Trx. This linkage is then repaired by another (resolving) Cys present in TrxR to regenerate the resting state Se–S species [124].

### 6.2. Carbon–Carbon

#### 6.2.1. Tyrosine–Tyrosine (Di-Tyrosine, Di-Tyr) Crosslinks

**Figure 5:**
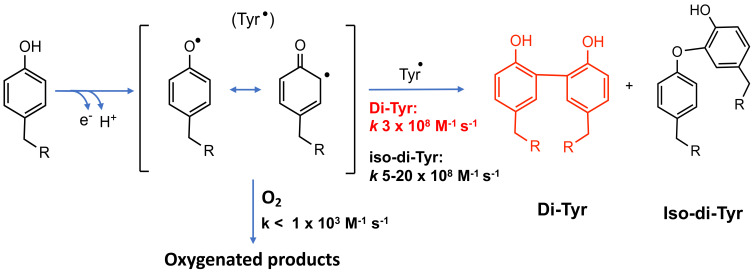
Formation and reactions of Tyr phenoxyl radicals (Tyr^•^). Tyr^•^ self-react to produce di-Tyr (o,o’-di-Tyr, red; iso-di-Tyr, black) or react with O_2_ to generate oxygenated products. Kinetic data from [130].

Di-Tyr crosslinks are formed deliberately by some enzymes, post-translationally, to give stability and elasticity to structural proteins (e.g., [125]). Nevertheless, protein exposure to oxidative environments, both biologically [126,127] and in food systems [128,129], can produce additional di-Tyr crosslinks. Thus, exposure of proteins to hydroxyl (HO^•^) and peroxyl (ROO^•^), peroxynitrous acid/peroxynitrite (ONOOH/ONOO^−^), nitrogen dioxide (NO_2_^•^), nitrosoperoxycarbonate (ONOOCO_2_^−^), carbonate (CO_3_^•−^), lipid hydroperoxides (LOOH) and photoinduced reactions can yield di-Tyr crosslinks [130,131,132]. These can be formed inter- or intramolecularly between two proteins, from two free Tyr, or between free Tyr and proteins, by radical–radical reactions involving two Tyr^•^ (Figure 5) [130,133]. Electron delocalization over the benzene ring and the phenolic oxygen results in the formation of two regio-isomers, one involving a carbon–oxygen bond (C3–O, iso-dityrosine, *vide infra*) and another with a carbon–carbon (C3–C3, *o,o’*) bond. The latter is thermodynamically preferred (Figure 5) [19,134,135]. The production of di-Tyr crosslinks is limited by alternative reactions, such as that with O_2_ (though this is slow, *k* < 1 × 10^3^ M^−1^ s^−1^, [130]), and also via fast reaction with other radicals such as O_2_^•−^ (which gives short-lived peroxides [37,136]) and nitric oxide (NO^•^) [137], and also with reducing agents such as ascorbate, thiols and polyphenols [138,139,140]. The extent of dimerization is also likely to be strongly modulated by steric and electronic interactions.

In pathophysiological contexts, di-Tyr crosslinking has been reported in low-density lipoproteins, arising from oxidative damage within human atherosclerotic lesions [61]; in erythrocytes exposed to a continuous flux of H_2_O_2_ [62]; in lens proteins, where it appears to originate from photo-oxidation of amino acids [63]; in tissues from a number of neurodegenerative conditions (e.g., amyloid-beta dimers in Alzheimer’s disease [64] and in α-synuclein [65,66], which may contribute to Parkinson’s disease); in lipofuscin pigments in the aged human brain [67]; in plasma of patients with chronic renal failure; and in urine of people with diabetes [68,69].

Di-Tyr crosslinks have also been detected on synthetic peptides exposed to peroxidase activity (e.g., hemoglobin and H_2_O_2_ [141]), and a large number of isolated proteins including (amongst others): lipoproteins [61,70,71], glucose 6-phosphate dehydrogenase (G6PDH) [53], ribonuclease A (RNAse A) [86], multiple extracellular matrix proteins [57,58,76], calmodulin [72], lysozyme [54,79], myoglobin [142,143], insulin [48,73,74] and human Δ25 centrin 2 [75].

The large body of evidence for di-Tyr crosslinks in oxidized proteins, fluids and tissues; the stability of this species (which is immune to reduction or repair); the association of this crosslink with the onset and development of human pathologies; and advances in analytical methods for the detection and quantification of di-Tyr (see below) have supported its use as a biomarker of protein oxidation in clinical studies [144,145].

Di-Tyr formation has also been reported in food systems, with both positive and negative effects. Protein crosslinking can give desirable textures and stability to processed foods, while negative effects have been reported with regard to poor protein digestibility and other undesirable properties [128,129]. Di-Tyr crosslinks have been detected in milk powders [146,147], during breadmaking [148], processing of myofibrillar proteins [149], in isolated caseins exposed to riboflavin-mediated photoreactions [51], and lactalbumin exposed to a laccase enzyme system [150] and UV-B light [50]. The presence of di-Tyr in foods, and consequent dietary intake, has resulted in studies on its toxicological properties. Intragastric administration of pure di-Tyr has been associated with metabolic alterations [151], including disrupted glucose metabolism [152], and renal alterations [153]. Interestingly, and of potential biological relevance, processed milks containing di-Tyr (and other Tyr oxidation products) can induce oxidative damage in plasma, liver and brain tissues, as well as spatial learning and memory impairments [154].

Di-Tyr crosslinks have also been detected in a wide variety of other organisms, including in insect cuticles, where di-Tyr crosslinks connect resilin proteins in a three-dimensional network [155]. Peroxidase-catalyzed di-Tyr crosslinks have also been detected in high concentrations in the oocyst walls of the apicomplexan parasite, *Eimeria maxima*, and in some bacterial spore proteins (e.g., those of *Bacillus subtilis*), with these providing a rigid framework that protects the oocyst or spores from harsh environmental conditions [60,77]. On the basis of these data, novel biomaterials have been developed that contain di-Tyr crosslinks that have excellent mechanical properties. Different strategies to induce efficient di-Tyr crosslink formation have been developed, including photoreactions mediated by ruthenium complexes and riboflavin [132].

#### 6.2.2. Tryptophan–Tryptophan (Di-Tryptophan, di-Trp) Crosslinks

**Figure 6:**
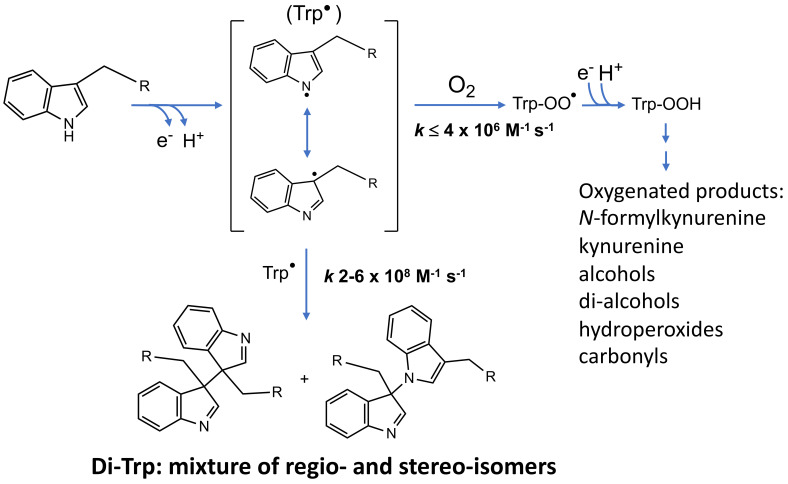
Formation and reactions of Trp indolyl radicals (Trp^•^). Self-reactions of Trp^•^ produce carbon–carbon (C3–C3) and carbon–nitrogen (C3–N1) di-Trp crosslinks. It should be noted that multiple stereoisomers are potentially formed for both di-Trp dimers. Kinetic constants for self-reactions of Trp^•^ and their reaction with O_2_ are from [159] and [36], respectively.

Trp is a major target for many oxidants, and a large number of oxidation products have been characterized [156]. Many of these involve the initial formation of (nitrogen-centered) indolyl radicals (Trp^•^) which, depending on the properties of the protein and environment, can self-react to generate di-Trp crosslinks (Figure 6), though less information is available on these species than di-Tyr. Di-Trp crosslinks have been reported on human superoxide dismutase 1 (hSOD1) upon exposure to H_2_O_2_ in the presence of carbonate ions [78], with the peroxidase activity of hSOD1 being responsible for the generation of CO_3_^•−^ that induce one-electron oxidation of the single, solvent-exposed, Trp32 residue in this protein and consequent intermolecular di-Trp crosslinking. This results in irreversible dimerization and is associated with non-amyloid protein aggregation and the occurrence of amyotrophic lateral sclerosis (ALS) [157]. CO_3_^•−^-mediated di-Trp crosslinks have also been reported for both free Trp [158] and lysozyme [79]. In the latter case, oxidation mediated by hSOD1 resulted in the detection of both hetero-dimers of lysozyme with hSOD1 and hSOD1 dimers [79]. These reports indicate a high propensity of CO_3_^•−^ to generate di-Trp crosslinks [79]. Di-Trp crosslinks have also been reported on lysozyme exposed to ONOOH/ONOO^−^ [159] and photo-oxidation mediated by kynurenic acid [160], as well as in solutions of free Trp and *N*-acetyl-Trp exposed to photo-reactions of riboflavin and kynurenic acid, respectively [161,162]. Di-Trp crosslinks have been also detected in lens proteins of patients with nuclear cataracts [80] and in α-crystallin proteins exposed to UV radiation and a photosensitizer [163].

Di-Trp crosslinks have also been detected in aggregates of α-lactalbumin (α-LA) exposed to UV-B light [50], and in FtsZ proteins of *M. jannaschii*, a thermophilic microorganism, exposed to peroxyl radicals [164]. The dimerization of Trp^•^ is fast (k ~ 2 − 6 × 10^8^ M^−1^ s^−1^ [159]), but dependent on the residue environment in peptides and proteins. Due to electron delocalization across the pyrrole and benzene rings, and the presence of chiral carbons, a complex mixture of regio- and stereo-isomers can be formed. This heterogeneity is likely to contribute to the limited number of reports on this type of crosslink, due to the difficulty in identifying and quantifying all of these species. This is exacerbated by a current absence of antibodies against this linkage (unlike di-Tyr, see below). Nonetheless, di-Trp crosslinks appear to be generated in low yields, probably due to the large number of alternative fates of Trp^•^ (Figure 6) and the low abundance of Trp in proteins (~1–2%). Despite the modest rate constant for addition of O_2_ to Trp^•^ (*k* ≤ 4 × 10^6^ M^−1^ s^−1^ [35,36]), oxygenated products and alternative crosslinks (e.g., Trp-Tyr, see below) are commonly detected, and at higher yields than di-Trp. Thus, only a small extent of the aggregation of α-LA induced by UV-B light was associated with di-Trp crosslinks, in contrast to high yields of disulfide bonds [50]. Nonetheless, under anaerobic conditions, or environments with low O_2_ levels (e.g., human lens [161]), the formation of di-Trp crosslinks appears to be favored.

The production of di-Trp crosslinks is also modulated by protein conformations, as reported for the extracellular matrix (ECM) protein fibronectin, when exposed to ONOO^−^/ONOOH [57]. Fibronectin is polymerized into elastic fibrils (fibrillation) at the cell surface, with this then allowing binding of other ECM components. This process is controlled (amongst others) by alterations to the protein conformation, with the compact structure present in plasma being resistant to fibrillation. However, an extended conformation generated by cell-generated forces (as well as by altered pH and salt concentrations) is more prone to fibrillation. MS analysis of tryptic peptides from fibronectin exposed to ONOO^−^/ONOOH showed that two of the four crosslinks detected correspond to di-Trp crosslinks [57]. One di-Trp crosslink (Trp445–Trp2264) was detected in both the compact and extended conformations, while another (Trp177–Trp2250) was only detected in the compact state. Interestingly, Trp2250 was oxidized to a high extent in the extended conformation, indicating that compact conformation of fibronectin favors radical–radical reactions (involving Trp177 and Trp2250), while the extended state favors other fates, including formation of 6-nitro-Trp [57].

Trp–Trp bonds have also been detected in the active sites of some enzymes, including amine dehydrogenases (e.g., methylamine dehydrogenase and aromatic amine dehydrogenases), where a tryptophan tryptophanylquinone crosslink is generated between C2 of an unmodified Trp residue, and C5 (on the benzene ring) of a Trp quinone (with the carbonyl groups present at C7 and C8) [165].

#### 6.2.3. Tyrosine–Tryptophan (Tyr–Trp) Crosslinks

Cross-reaction between Tyr^•^ and Trp^•^ can lead to Tyr–Trp crosslinks. These species are less well characterized than those described above, and the precise structure of these species remains to be elucidated (i.e., the nature of the crosslink bond and sites on the two rings that are joined). MS analyses of solutions containing free Tyr and Trp incubated with CO_3_^•^**^−^** have provided evidence for three different, isomeric, Tyr–Trp crosslinks, with these likely to involve carbon–carbon bonds, and probably between the ortho (C3) position on the Tyr ring, and C3 on the indole ring of Trp [158].

Tyr–Trp crosslinks have been detected in peptides and isolated proteins exposed to multiple oxidants, including a cytochrome c peroxidase mutant exposed to a heme iron-peroxide reaction [81]; in glucose-6-phosphate dehydrogenase (G6PDH) exposed to ROO^•^ [53]; model peptides exposed to pulsed UV light [166]; in lysozyme treated with ROO^•^ and exposed to photooxidation reactions mediated by riboflavin and rose Bengal [54,55,56]; in fibronectin exposed to ONOOH/ONOO^−^ [57]; in protein extracts of the Gram-positive bacterium *Lactococcus lactis* exposed to photooxidation [167]; and proteins extracted from human cataractous lenses [80].

#### 6.2.4. Other Carbon–Carbon Crosslinks

As most protein carbon radicals, P^•^, react rapidly with O_2_ (see above), termination reactions between two P^•^ are limited under most biological conditions. The formation of some P–P crosslinks has, however, been reported in model peptide systems exposed to radiation-induced radicals [33], and similar species may be formed in biological situations where the pO_2_ value is low (e.g., in solid tumors, tissues subject to hypoxia). This requires further study.

### 6.3. Carbon–Nitrogen (C–N) Crosslinks

A significant number of carbon–nitrogen crosslinks have been identified, with the majority of these arising from molecule–molecule reactions, with the nitrogen atom acting as a nucleophile, though a small number of radical–radical crosslinks have been characterized.

Delocalization of the unpaired electron over the aromatic rings of Tyr^•^ and Trp^•^, followed by radical–radical reactions has been proposed, on the basis of MS data, to give di-Trp species with carbon–nitrogen (C3-N1) bonds (Figure 6) [158]. The exact structures of these species are not completely clarified, though it is clear that multiple isobaric species are formed [159,161]. Some of these crosslinks appear to be of pathological relevance, as they have been detected on lens crystallin proteins subjected to photo-oxidation [161] and in human cataractous lenses [80].

The much larger group of molecule–molecule reactions that generate C–N crosslinks primarily involve nucleophilic reactions of the nitrogen centers on Lys and hydroxy-Lys (N^ε^-amine), Arg (guanidine nitrogen) and His (imidazole nitrogen) with either the carbon atom of carbonyl functions (Schiff base reactions) or other electron-deficient carbon centers (via Michael addition). For the former type of reaction, the carbonyl can be formed enzymatically (e.g., by LOX and LOXL enzymes, see earlier) or as a result of oxidation of alcohols (Ser/Thr) or C–H bonds in the presence of O_2_ (e.g., via ROO^•^ and ROOH [31,168]).

In the case of the Cu^2+^-dependent LOX/LOXL reactions and collagen crosslinking, the initial Schiff base adducts can undergo multiple further condensation reactions that allow several chains to be linked together via a single site [11]. Thus, there is abundant evidence for lysyl pyrrole, hydroxylysyl pyrrole, lysyl pyridinoline and hydroxylysyl pyridinoline species involving three or four collagen chains [169]. The mechanism of formation of these species involves the initial formation of a two-chain crosslink and then further condensation with a Lys/hydroxy-Lys on third and fourth chains. These species are critical to the correct assembly of collagen-containing extracellular matrices in tissues [11].

Similar reactions occur with (Ca^2+^-dependent) transglutaminases that form isopeptide bonds between the Lys side-chain amine and amide (Gln, Asn) or carboxylate (Asp) residues, with the amide/carboxylate ‘activated’ via reaction with the Cys residue of a Cys–His–Asp catalytic triad on the enzyme. The reaction of the carbonyl function with the Cys residue generates a thioester that is then susceptible to reaction with the Lys amine [22,23,24]. Such isopeptide bonds are highly resistant to most proteases and are widely encountered in the formation of insoluble protein polymers/aggregates, such as those found in brain tissue lesions of people with Alzheimer’s disease [24,170], in some bacterial spore proteins [60] and in the food industry, where these reactions are used as glues to alter food texture and properties [171,172].

**Figure 7:**
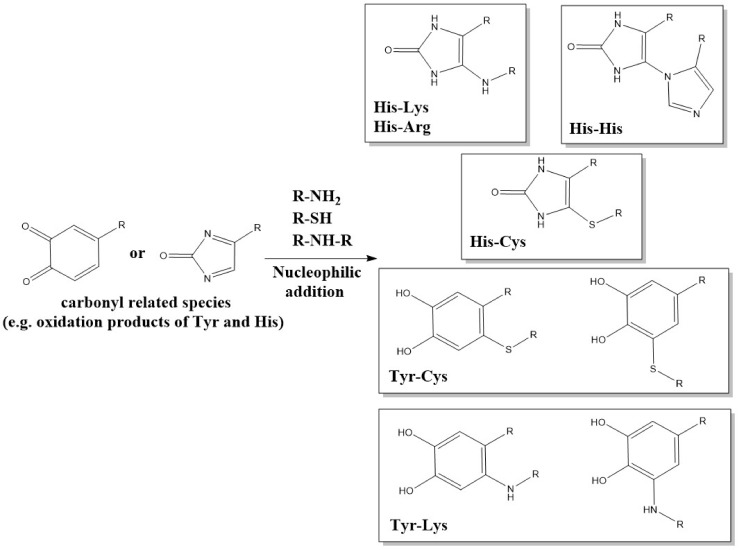
Michael addition reactions of amino acid side-chains to oxidized His and Tyr residues.

Reactions also occur with α,β-unsaturated aldehyde and ketones and related species via Michael addition reactions (Figure 7). Thus, intra- and interchain crosslinks can be generated by the reaction of Lys/Arg/His residues with an oxidized imidazole group of the amino acid His, which acts as a Michael acceptor. These Lys–His, Arg–His and His–His products have been detected on multiple proteins, and particularly those subjected to photo-oxidation, where His residues are a major initial target of damage (e.g., [82,84,85,86,173]). They have also been detected in antibodies exposed to (photo)oxidation [82,84]. His-containing crosslinks have also been reported in some collagens, with these probably formed by nucleophilic attack of an imidazole nitrogen on an α,β-unsaturated structure formed via other crosslinking reactions [174].

Related Michael reactions occur with Tyr-derived quinones formed on oxidation of Tyr residues, with nitrogen nucleophiles, such as the N^ε^-amine of Lys side-chains undergoing adduction reactions with the oxidized ring (Figure 7). These reactions occur with lower rate constants than the corresponding reaction with the RS^−^ from Cys [110,175] (i.e., C–S crosslinks predominate over C–N linkages, through this is dependent on the availability and abundance of Cys versus Lys residues, with the latter usually predominating numerically). These reactions are important in the coupling of proteins, including via N-terminal amine groups, by tyrosinase and polyphenol oxidases (e.g., [176]) and in the formation of insect exoskeletons/cuticles of many insects [177], as well as the ‘glues’ that attach mussels to rocks [178,179].

### 6.4. Carbon–Oxygen

As alcohols (ROH) are poorer nucleophiles when compared to RS^−^ and neutral amines (RNH_2_), there are limited numbers of known carbon–oxygen crosslinks formed via molecular processes such as Michael reactions. However, such coupling can occur via radical–radical reactions, with the most well-established example being the formation of iso-di-tyrosine (iso-di-Tyr), where two Tyr^•^ dimerize via the phenolate oxygen of one ring, and C3 on the second ring (Figure 5); this appears to be a minor reaction compared to carbon–carbon coupling (see above) [19]. Iso-di-Tyr crosslinks have been reported in the extensin proteins that contribute to the architecture of wall plant cells [180], and in systems exposed to the myeloperoxidase–H_2_O_2_ system of human phagocytes, suggesting that these species may be present in tissues subject to acute or chronic inflammation, such as atherosclerosis [19].

## 7. Secondary Reactions of Crosslinks

In most biological systems, protein crosslinks, and particularly the formation of irreversible covalent crosslinks such as di-Tyr, di-Trp and Tyr–Trp, are considered as ‘final’ oxidation products [181]. However, over-oxidation of these species is possible, particularly under conditions of extensive oxidative damage, or under environments with long-term protein exposure to oxidants, where secondary one-electron oxidation with formation of radicals such as di-Tyr^•^, di-Trp^•^, or Tyr–Trp^•^ may occur. Such radicals can mediate similar reactions to those described above for Tyr^•^ and Trp^•^, including reaction with O_2_ to produce oxygenated products (e.g., alcohols and hydroperoxides) and self-reactions to generate trimers and oligomers. Thus, formation of tri-Tyr and pulcherosine crosslinks have been detected in human phagocytes [19], while di-, tri- and tetra-Tyr have been reported in structural proteins of plant parasitic nematodes [182]. In addition, oligomers of Tyr (*n* = 2–8) have been reported in α-lactalbumin exposed to a horseradish peroxidase–H_2_O_2_ system [183]. Tri-Trp has been reported in trimers of hSOD1 triggered by CO_3_^•^**^−^** [78], while tri-Trp and a di-Trp hydroperoxide (di-Trp-OOH) were reported in solutions of free Trp and riboflavin illuminated with a high-intensity 365 nm light-emitting diode [162].

In contrast, photo-oxidation (at 320 nm) of di-Tyr, in the presence of O_2_, has been reported to occur via processes involving O_2_^•^**^−^**, singlet oxygen (^1^O_2_) and H_2_O_2_ [184]. These observations were ascribed to the action of di-Tyr crosslinks as photosensitizers that could induce photo-damage to other biomolecules [184]. These findings suggest that di-Tyr crosslinks may be able to extend oxidation processes, opening new pathways of reactions, though these are only likely to be of major impact in systems with very extensive extents of oxidation. The scope and role of these pathways is unexplored, as well as the ability of peroxides such as di-Trp-OOH to extend protein oxidation (in line with the capacity of other hydroperoxides [31]).

## 8. Detection of Crosslinks, including Advantages and Disadvantages of Different Methods

**Figure 8:**
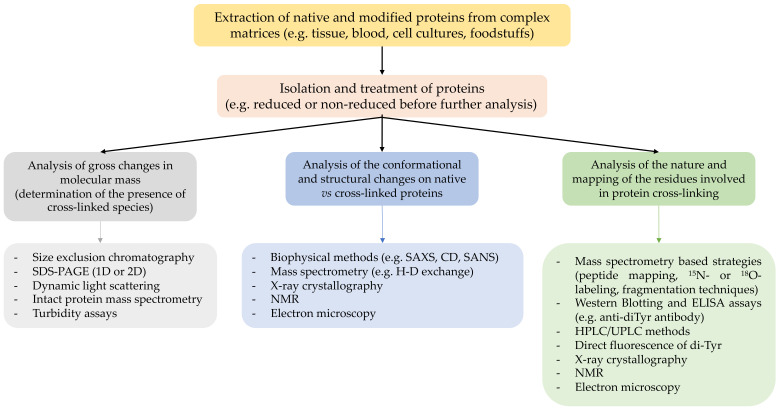
Overview of methods to detect and characterize crosslinked proteins and the sites/types of modifications. Abbreviations used: CD: circular dichroism, SANS: small angle neutron scattering, SAXS: small angle X-ray scattering, H–D: hydrogen–deuterium exchange mass spectrometry.

Despite considerable methodological advancements, analysis of intra- or intermolecular protein crosslinks remains a challenging task, and the mechanisms underlying the formation of some known crosslinks remain to be established. There are also, undoubtedly, more types of crosslinks that remain to be discovered. This lack of knowledge arises partly due to the complexity of some structures, and the relatively poor current ‘tool-box’ to detect and predict the chemistry of the species and the sites involved. The following section therefore provides an overview of current approaches to the identification and characterization of oxidation-induced crosslinks, with a focus on both mass spectrometry (MS) and techniques (e.g., UPLC, immunological methods) that provide complementary information. It is clearly advisable to use combinations of methods and to investigate both gross modifications (e.g., changes in the molecular mass) and structural/conformational changes in crosslinked vs. native proteins with methods that allow the chemical nature and the residues involved in the formation of these species to be determined (Figure 8). Each of these methods has individual advantages and disadvantages.

### 8.1. Analysis of Changes in Molecular Mass by Electrophoresis and Size Exclusion Chromatography (SEC)

Protein electrophoresis (e.g., SDS-PAGE) and SEC-derived methodologies are excellent tools to assess the presence of crosslinked proteins in samples. Separation by electrophoresis is typically achieved through the use of polyacrylamide gels with different pore sizes. Diverse strategies can be utilized with this approach, such as running gels under native, denaturing and/or reducing conditions. This allows the investigation and differentiation of the contributions of reducible intermolecular bonds (e.g., disulfides between two protein chains) from non-reducible bonds (e.g., carbon–carbon bonds). Similarly, SEC can separate and fractionate soluble proteins based on their hydrodynamic radius and molecular mass, and depending on the mobile phase used, it can also provide information about the nature of the crosslinks (i.e., reducible or non-reducible intermolecular bonds).

Whilst these techniques can provide information on the presence of *inter*molecular bonds, they rarely provide information about the presence of *intra*molecular species. Moreover, data analysis needs to be carried out with care, as multiple proteins may be present in each band/fraction from complex matrices. This complexity can be overcome by using two-dimensional gel electrophoresis, with proteins separated firstly on the basis of their isoelectric point, and subsequently by molecular mass. Both methodologies have been successfully employed in combination with MS-based strategies [54,56,80,84,93,94,141,185], with isolation and enrichment of fractions containing dimers (and species with higher degrees of oligomerization) from non-crosslinked monomers before MS analysis. This approach has been used successfully to characterize crosslinked forms of IgG, α-synuclein and lysozyme [56,84,141]. Two-dimensional gel electrophoresis can also be used for specific detection of proteins connected by intermolecular disulfides [185]. In this approach, the proteins are first separated by non-reducing SDS-PAGE in the first dimension, and then separated by SDS-PAGE under reducing conditions in the second dimension. Proteins lacking intermolecular disulfide bonds line up on the diagonal, whereas proteins connected by intermolecular disulfide bonds dissociate from each other in the second dimension and migrate below the diagonal.

### 8.2. Analysis of Protein Crosslinks by Western (Immuno-) Blotting and ELISA Assays

The use of antibodies to investigate the formation of oxidation products, including di-Tyr, is a widely used strategy. These are typically examined using immunoblotting or ELISA assays, with the former providing (limited) information on the nature and identity of the proteins on which the crosslinks are present, and whether these are intramolecular (in a monomer) or interchain species. However, there are few well-characterized antibodies against crosslinked species, and these vary significantly in their specificity and selectivity, with some having significant cross-reactivity with other materials. Furthermore, crosslinks buried within highly aggregated species may be poorly, or not, recognized by (large) antibodies. Thus, appropriate control experiments are critical, and both positive and negative data should be validated by alternative methods.

Despite these caveats, immunoblotting has been widely used to detect di-Tyr present in α-synuclein in Lewy Bodies in Parkinson’s disease [186], in atherosclerotic plaques [187], and on multiple isolated proteins, including tropoelastin, α-synuclein, caseins, glucose-6-phosphate dehydrogenase, laminins and fibronectin [51,53,58,65,76,188]. Relative yields (i.e., versus the parent) can be achieved by ELISA, and this can serve as a very useful screening tool.

### 8.3. Direct Detection by Spectrophotometric and Fluorometric Assays

Some crosslinked species, as well as heavily aggregated proteins, can be monitored by spectrophotometry and fluorescence spectroscopy. For example, turbidity changes in solutions can be used (in an approximate manner) to monitor the time-course of protein crosslinking and aggregation [189,190]. However, this approach does not provide information on the type and nature of the crosslinked proteins and is useful only when there is extensive formation of heavily aggregated species, as soluble dimers and oligomers do not contribute significantly to the turbidity of solutions.

In contrast, fluorescence experiments can provide relatively specific data on crosslinks such as di-Tyr (excitation and emission maximum at ~280 and ~410 nm, respectively) [191]. Such data need to be interpreted with care, particularly with intact proteins or complex systems, as other fluorescent or optically absorbing species (e.g., Tyr, Trp, Trp-derived products, co-factors) may be present that distort excitation or emission processes. Thus, depending on the sample complexity, di-Tyr detection by fluorescence can provide useful information, but requires further confirmation using other methodologies. In this context, coupling fluorescence detection methods with HPLC, UPLC or GC separation (see below) has proven to be a valuable strategy to detect and quantify di-Tyr in mixtures of amino acids and oxidation products arising from acid hydrolysis of complex protein and tissue samples [191,192].

### 8.4. HPLC/UPLC Methodologies

These techniques can provide important quantitative data on both the consumption of the parent amino acid residues, and product formation, including Trp- and Tyr-derived crosslinks [158,191,193,194]. Before analysis, proteins are often isolated by precipitation (e.g., from homogenates or cell lysates) using acids (e.g., trichloroacetic acid) or organic solvents (e.g., ice-cold acetone or ethanol), followed by sample clean-up (e.g., delipidation), and then hydrolysis (with acid, base or enzymes) to give free amino acids and products. The advantages and disadvantages of this approach have been recently reviewed in [192], and therefore, the following is focused solely on the detection and quantification of crosslinked species.

The free amino acids and crosslinked products obtained from hydrolysis are separated by HPLC/UPLC and can be quantified by MS [158,159,193], by fluorescence detection (directly for di-Tyr [51], or by pre-column tagging for parent amino acids using sensitive fluorescent tags such as *o*-phthaldialdehyde [191]), UV absorption, or, for some species, electrochemical oxidation [191,195]. For HPLC/UPLC methodologies coupled to MS detection, heavy atom labeling (usually ^2^H, ^13^C, or ^15^N) can be utilized for accurate quantification [193]. Unfortunately, these approaches do not usually provide information on the *sites* of modification within proteins, or information on which proteins they were located on, if multiple species were present. This drawback, along with the limited number of crosslinked species that can be detected and quantified using these methods (di-Tyr, di-Trp, Trp-Tyr and cystine), makes it sensible to combine this approach with other methods (e.g., peptide mass mapping or immunoblotting).

### 8.5. Detection and Characterization of Crosslinked Proteins Using Other Biophysical Approaches

Biophysical techniques including circular dichroism (CD), light scattering, small angle neutron scattering (SANS), small angle X-ray scattering (SAXS), X-ray crystallography, NMR spectroscopy, and electron microscopy can provide useful information on protein structure. These methods are sensitive to modified structures, supplying valuable information on changes on morphology (i.e., mass, size and shape), secondary structure and solubility [45,196,197]. Some of these can also yield data on increased electron density between residues, thus supporting the presence of both intra- and intermolecular crosslinked species. This approach has been used in X-ray crystallographic studies, to determine the exact sites of crosslinks in oxidized peroxiredoxin 5, thioredoxin 2 and γS-crystallin, and to elucidate a covalent crosslink between Cys and Lys containing an N–O–S bridge [45,197,198,199]. However, most of these methods (with the exception of X-ray crystallography, NMR spectroscopy and cryogenic electron microscopy) cannot provide a structure of sufficiently high-resolution to provide definitive identifications and they must therefore be combined with other methodologies. Moreover, these methods are currently limited to homogeneous (single protein) samples that are available in large quantities (mg amounts).

### 8.6. Mass Spectrometry (MS)-Based Detection and Structural Characterization of Crosslinked Proteins

MS is a highly versatile technique for analysis of protein crosslinks that can be applied to (i) detect crosslinks and quantify their abundance, (ii) localize the specific crosslinking sites within polypeptides and (iii) reveal the identity of the crosslinked proteins. All of these questions cannot, however, be readily answered in a single experiment, and careful consideration must be given to appropriate workflows for specific applications.

#### 8.6.1. MS Analysis of Crosslinked Amino Acids

Crosslinked amino acids can be detected and quantified by GC-MS or LC-MS, with the latter being most commonly used as it limits the need for derivatization and high-temperature conditions. Free crosslinked amino acids (i.e., not protein-bound) such as di-Tyr can be detected in body fluids such as urine, plasma and cerebrospinal fluid [200,201]. As outlined above, crosslinked amino acids can also be released from proteins either through acidic or alkaline hydrolysis, or through the use of unspecific proteases (e.g., pronase that hydrolyses proteins to single amino acids) [61,66,186]. The choice of hydrolysis method depends on the chemical stability of the investigated crosslink. For example, di-Trp crosslinks are unstable to acid hydrolysis (as the indole ring undergoes acid-catalyzed cleavage) and therefore, alkaline hydrolysis or pronase digestion is preferred [159].

#### 8.6.2. MS Analysis of Crosslinked Peptides

Degradation of proteins using specific proteases such as trypsin releases peptides that can be analyzed by MS. This is the principle behind protein identification in standard ‘bottom-up’ proteomics, where the experimental mass-to-charge (*m/z*) values for the experimentally generated peptides are compared to theoretical values based on the known specificity of the enzyme. This type of workflow can also be applied for the identification of crosslinked peptides, since the mass of the crosslinked peptides can be predicted (for example, di-Tyr yields a mass shift of −2 Da relative to the sum of the two crosslinked peptides). The analysis of crosslinked peptides is, however, often more challenging, since the mass spectra of crosslinked peptides are more complex than those from regular (non-crosslinked) peptides. This is mainly due to the fact that the MS/MS spectra of crosslinked peptides may contain fragments from both of the linked individual peptides. Furthermore, the number of potential combinations of crosslinked peptides to take into consideration may be very large due to the complex chemistry of oxidation-induced crosslinking. This is particularly problematic if complex (cell/tissue) samples are analyzed and if intermolecular crosslinks are considered in database searches against large proteins databases (e.g., the entire human proteome). Due to these issues, there is a significant risk of false positive identifications, and it is therefore important to carefully validate the data, for example, using an ^18^O-labeling approach. This strategy relies on the ability of trypsin (and other related proteases) to incorporate two ^18^O atoms from isotope-labeled water molecules into each C-termini of proteolytic peptides [202]. Since crosslinked peptides contain two C-termini, a total of four ^18^O atoms will be incorporated instead of two ^18^O for regular (non-crosslinked peptides). Thus, it is possible to distinguish crosslinked peptides from non-crosslinked peptides based on their isotope labeling pattern [167,203]. This strategy has been successfully applied to validate oxidation-induced crosslinks in a wide range of proteins, including glucose-6-phosphate dehydrogenase, lysozyme, RNase A, elastin, fibronectin, cyclic AMP receptor protein, C-reactive protein and β-2-microglobulin (B2M) [53,54,55,56,57,59,86,93,94,203].

Identification of crosslinked peptides can also be facilitated by MS-based fragmentation of the crosslink bond, yielding individual mass spectra of the two formerly linked peptides, which can be analyzed separately. For stable crosslinks such as di-Tyr, this has recently been accomplished by ultraviolet photodissociation (UVPD) fragmentation [141]. For more labile crosslinks, such as disulfides, in-source fragmentation can be utilized [204,205]. Disulfides can also be probed indirectly through MS analysis of alkylated Cys thiol groups released after in vitro disulfide reduction using chemical (e.g., dithiothreitol) or enzymatic (e.g., thioredoxin [206]) treatment.

## 9. Crosslink Quantification

At present, there are few methods that allow absolute quantification of crosslink concentrations, with a major limitation being the non-availability of pure standards, particularly from commercial sources. Thus, there is a pressing need for further pure crosslink standards for quantitative analyses. Disulfides are a major exception, together with di-Tyr (which is commercially available) and a few species generated via glycation reactions (e.g., pentosidine). Di-Tyr can therefore be quantified in absolute terms using some of the methods outlined above, using the purified material to construct standard curves (e.g., for MS or fluorescence detection, and UPLC/LC separation). Relative concentrations can also be obtained from ELISA assays using an anti-di-Tyr antibody, and approximate concentrations from MS analyses. In nearly all cases, even with isolated proteins where protein turnover is not a confounding factor, the absolute concentrations determined by these approaches give low values, suggesting that there are many other important crosslinks which are either undetected, or which we cannot quantify.

## 10. Future Perspectives

Irreversible protein crosslinks have been associated with the onset and development of pathological conditions and human diseases. Similarly, reversible crosslinks, such as disulfides, appear to play a key role in cell signaling events, primarily as a result of reversible thiol–disulfide switches or related species (e.g., in the case of protein tyrosine phosphatase-1B, PTP-1B). The growing realization of the importance of these events has increased interest in the detection and quantification of these species together with irreversible crosslinks and other protein oxidation products (such as 3-nitroTyr, 3-chloroTyr, etc.) as biomarkers of biological events. This includes both physiological signaling and the assessment of the extent of oxidative damage for clinical purposes, such as diagnosis, prognosis, prevention or decisions on therapies/treatments. Di-Tyr crosslinks are recognized as reliable biomarkers of several pathologies, particularly neurodegenerative disorders [144]. However, this species only represents a fraction of the total pool of covalent crosslinks generated when proteins are exposed to oxidative conditions. As a consequence, new investigations aimed at understanding the role that this and other crosslinks (e.g., di-Trp or Tyr–Trp) play in controlling biological activity and contributing to human disease, and their use as clinical biomarkers, are necessary. In particular, it will be of interest and importance to determine how individual crosslinks modulate protein lifetime and turnover (e.g., by proteasomes). Current data do not typically allow such an assessment, as the methods used to generate crosslinks also induce multiple other alterations on the proteins, prohibiting the identification of cause and effect relationships. Studies focused on the mechanisms underlying crosslink formation in biological systems as well as the analytical strategies for their detection and quantification should also be considered. Such investigations are also likely to be valuable in other contexts where oxidation is relevant, such as in the processes triggered by physical exercise [207]; in food processing, formulation and degradation; in determining the shelf-life of protein-based medicines and therapeutics; and the development of new biomaterials.

## Figures and Tables

**Table 1:** Examples of major non-disulfide protein crosslinks generated during non-enzymatic oxidative processes and methodologies employed to characterize them.

Crosslinked Residues	Protein(s)	Chemical Nature and/or Mechanism of Formation of the Crosslink	Method(s)	Refs
Tyr-Cys	a) Myoglobin b) Galactose oxidase c) Cysteine dioxygenase	1) Michael addition from thiols (Cys) to oxidized Tyr species (a) 2) Thioether bridge (C-S) (b and c)	Mass spectrometry (a) X-ray crystallography (b, c)	[42,43,44]
Trp-Cys	Human growth hormone (hGH)	1) Michael addition from N (Trp indole) to DHA (formed from Cys) 2) Thioether bridge (C-S)	Mass spectrometry	[41]
Met-Hydroxy-lysine	Collagen IV	Formation of S=N bridge (sulfilimine bond) induced by peroxidasin/HOBr	Mass spectrometry	[17]
Lys-Cys	Transaldolase	Nitrogen–oxygen–sulfur (NOS) link/redox switch	X-ray crystallography	[45]
Cys-Ser	a) Human growth hormone b) Tyrosine phosphatase 1B	1) Formation of a vinyl ether between Ser and Cys that result in the elimination of the thiol group from Cys (a) 2) Sulfenyl amide (S–N bridge) between Cys-OH and main-chain amide of Ser residue (b)	Mass spectrometry (a) X-ray crystallography (b)	[41,46]
Cys-Phe	hGH	Crosslink between thioaldehyde from Cys and dehydrophenylalanine generated from Phe	Mass spectrometry	[41]
Cys-DHA Cys-DHB	Lens proteins (βB1, βB2, βA3, βA4 and γS crystallins)	Nucleophilic addition from Cys (GSH) to DHA or DHB	Mass spectrometry	[47]
Tyr-Gly	Insulin	Michael addition of primary amines (N-terminal Gly) to oxidized Tyr species	Mass spectrometry	[48]
Trp-Gly	Matrilysin (Matrix metalloproteinase 7)	Crosslink between 3-chloroindolenine (3-Cl-Trp) and the main-chain amide adjacent to a Gly	NMR spectroscopy	[49]
Tyr-His	Insulin	Michael addition from His to oxidized Tyr	Mass spectrometry	[48]
Tyr-Tyr (selected data)	Isolated proteins including: α-lactalbumin, caseins, glucose 6-phosphate dehydrogenase, lysozyme, fibronectin, laminins, tropoelastin, cAMP receptor protein, α-synuclein, calmodulin, insulins, hemoglobin, human Δ25 centrin 2. Human lipoproteins Human plasma proteins, including those from people with chronic renal failure Human atherosclerotic lesions Erythrocytes exposed to H_2_O_2_ Brain proteins (amyloid-beta and α-synuclein) from Alzheimer’s subjects Lipofuscin from aged human brain Urine from people with diabetes Human lens proteins Bacterial spore coat proteins Parasite oocysts	C–C and/or C–O crosslinks via radical–radical reactions	Western blotting UPLC/HPLC with various detection methods Mass spectrometry	[48,50,51,52,53,54,55,56,57,58,59,60,61,62,63,64,65,66,67,68,69,70,71,72,73,74,75,76,77]
Trp-Trp	a) α-Lactalbumin b) Superoxide dismutase 1 (hSOD) c) Lysozyme-hSOD d) αB-Crystallin e) Fibronectin	C–C or C–N crosslinks via radical–radical reactions	Mass spectrometry	[50,57,78,79,80]
Tyr-Trp	a) Cytochrome c peroxidase b) α-Lactalbumin c) Glucose 6-phosphate dehydrogenase d) Lysozyme e) β-Crystallin f) Human cataractous lenses g) Fibronectin	C–C (or C–O and C–N) crosslinks via radical–radical reactions	X-ray crystallography (a) Mass spectrometry (b–g)	[50,53,56,57,80,81]
His-His	a) Immunoglobulin G1 b) Immunoglobulin G4 c) *N*-Ac-His	Nucleophilic addition of His to oxidized His	Mass spectrometry (a,b) NMR (c)	[82,83,84,85]
His-Arg	Ribonuclease A (RNAse)	Nucleophilic addition of Arg to oxidized His	Mass spectrometry	[86]
His-Lys	Immunoglobulin G1	Nucleophilic addition of Lys to oxidized His	Mass spectrometry	[82,84]
His-Cys	Immunoglobulin G1	Nucleophilic addition of Cys to oxidized His	Mass spectrometry	[84]
Tyr-Lys	a) RNAse b) Interferon beta-1a c) Insulin	Michael addition of Lys to oxidized Tyr	Mass spectrometry	[48,86,87]

## Abbreviations

ALS	amyotrophic lateral sclerosis
α-LA	α-lactalbumin
CD	circular dichroism
CTQ	cysteine tryptophanylquinone
DHA	dehydroalanine
DHB	dehydrobutyric acid
Di-Trp	di-tryptophan
Di-Trp-OOH	di-tryptophan hydroperoxides
Di-Tyr	di-tyrosine
DOPA	3,4-hydroxyphenylalanine
ELISA	enzyme-linked immunoassay;
ECM	extracellular matrix
FtsZ	filamenting temperature-sensitive mutant Z
GC-MS	gas chromatography mass spectrometry
G6PDH	glucose 6-phosphate dehydrogenase
GPx’s	glutathione peroxidases
GSA	glutathione sulfonamide
GSH	glutathione
GSSG	glutathione disulfide
H_2_O_2_	hydrogen peroxide
HOBr	the physiological mixture of hypobromous acid and its anion, hypobromite
HOCl	the physiological mixture of hypochlorous acid and its anion, hypochlorite
HPLC	high performance liquid chromatography
hSOD1	human superoxide dismutase 1
Iso-di-Tyr	iso-di-tyrosine
LC-MS	liquid chromatography mass spectrometry
LDLR	low-density-lipoprotein receptor
LOOH	lipid hydroperoxide
LOX	lysyl oxidase
LOXL	lysyl oxidase-like enzyme
MS	mass spectrometry
NMR	nuclear magnetic resonance
^1^O_2_	singlet oxygen
ONOO^−^/ONOOH	the physiological mixture of peroxynitrite anion and peroxynitrous acid
P^•^	protein carbon-centered radical
POO^•^	protein peroxyl radical
P-P	protein dimers
PTP1B	protein tyrosine phosphatase 1B
RS^•^	thiyl radical RS
RS^−^	thiolate anion
R-NHBr	bromamines
R-NHCl	chloramines
ROH	alcohol
ROOH	alkyl hydroperoxide
RNAse A	ribonuclease A
R-NH_2_	neutral amine
RS-NHR′	sulfenamide
RSNO	S-nitroso thiol
RSOH	sulfenic acid
RSO_2_H	sulfinic acid
RSO_3_H	sulfonic acid
RS-(O)-NHR′	sulfinamide
RS-(O)_2_-NHR′	sulfonamide
RS-X	sulfenyl halide
SANS	small angle neutron scattering
SAXS	small angle X-ray scattering
SDS-PAGE	sodium dodecyl sulphate–polyacrylamide gel electrophoresis
SEC	size exclusion chromatography
SUMOs	small-ubiquitin-like modifier proteins
Tri-Trp	tri-tryptophan
Trp^•^	tryptophan indolyl radical
Trx	thioredoxin
TrxR	thioredoxin reductase
TTQ	tryptophan-tryptophanylquinone
Tyr^•^	tyrosine phenoxyl radical
UPLC	ultra-performance liquid chromatography
UVPD	ultraviolet photodissociation
